# Efficacy and safety of microwave ablation in solitary kidney patients with T1a small renal masses

**DOI:** 10.1007/s00261-024-04779-7

**Published:** 2025-01-07

**Authors:** Carlos Justo-Jaume, Max Stempel, Jessica Qiu, Luis Gonzalez Miranda, Guofen Yan, Genevieve Lyons, Kenneth Sands, Noah Schenkman, Tracey Krupski, Stephen Culp, Jennifer Lobo

**Affiliations:** University of Virginia, Charlottesville, USA

**Keywords:** Renal cell carcinoma, Solitary kidney, Microwave ablation, Kidney function, Clinical outcomes

## Abstract

**Objectives:**

To assess the safety and efficacy of using microwave ablation (MWA) to treat solitary kidney (SK) patients with T1a renal cell carcinoma (RCC).

**Methods:**

Retrospective analysis of a prospectively maintained database identified patients with T1a RCC with either congenital or acquired SK. Estimated glomerular filtration rate (eGFR) was calculated from serum creatinine before MWA and after at 6 and 12 months post-procedure. The local recurrence-free survival (LRFS), metastatic-recurrence free survival (MRFS), cancer-specific survival, and overall survival were analyzed with the Kaplan–Meier method.

**Results:**

A total of 26 patients met inclusion criteria, including 3 congenital and 23 acquired SK patients. eGFR was lower at both 6 and 12 months post-procedure compared to pre-procedure, with the congenital SK group having a more pronounced reduction in eGFR at both post-procedure timepoints. Median follow-up time was 28.6 months (IQR 12.4–55.4). Four patients (15.4%) experienced local recurrence. For LRFS, mean survival time was 69.4 months. There were 5 patients (19.2%) that experienced metastatic recurrence, with median and mean survival time at 101.1 and 82.0 months, respectively. The mean time for RCC-specific survival was 94.7 months, while median and mean time for overall survival was 43.1 and 61.7 months, respectively.

**Conclusion:**

With a moderate reduction in renal function and a comparable rate of local recurrence compared to prior literature, this work demonstrates that MWA remains a viable alternative to more invasive techniques, particularly for high-risk SK patients with RCC. Our work highlights the need for further research on effectiveness of MWA in cancer control and preservation of renal function in larger cohorts of SK patients over extended follow-up times.

## Introduction

Partial nephrectomy (PN) is the gold standard surgical treatment for cT1a small renal masses (SRMs), but in solitary kidney (SK) patients, PN may further risk damaging the SK and worsening renal function [1]. Balancing the morbidity of kidney function decline and mortality of RCC is difficult in SK patients. Thermal ablative treatments are being used increasingly for cT1a masses in select patient populations with emerging data demonstrating equivalent oncological outcomes [2, 3].

One promising alternative to PN is microwave ablation (MWA), which targets the tumor specifically and avoids warm ischemia time, potentially preserving more renal parenchyma tissue compared to partial nephrectomy [2]. Our institution uses MWA compared to other ablative techniques for treating SRMs due to improved ease of use and predictability. Small cohort studies have shown that cryoablation (CA), radiofrequency ablation (RA), and MWA are safe and effective in SK patients with RCC [4–9]. However, there is no consensus in the field on the role of MWA therapy in SK patients with RCC. In this study, we build on prior literature by assessing the safety and effectiveness of MWA in a cohort of patients with congenital and acquired SK based on renal function and oncological outcomes.

## Methods

### Patient selection

**Fig. 1:**
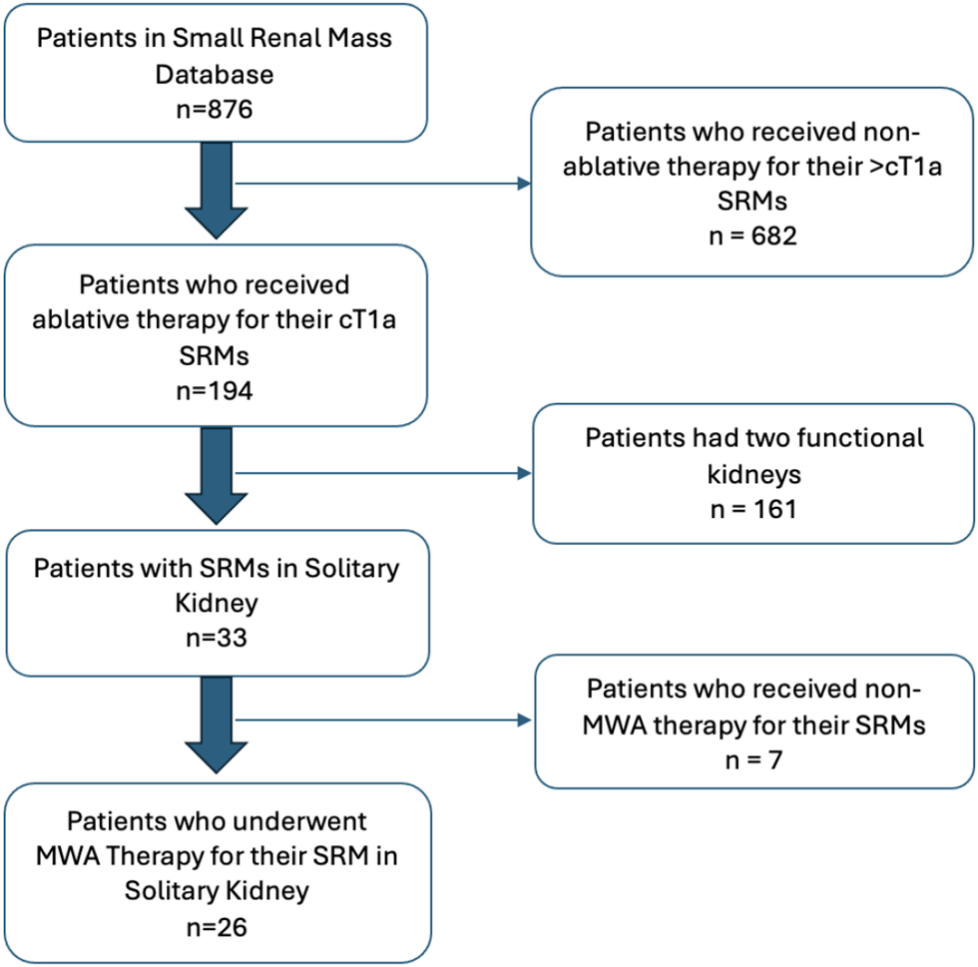
Criteria used for including and excluding patients. MWA: microwave ablation, SRMs: small renal masses, cT1a: clinical T1a

Our SRM database, approved by the institutional review board, is stored in REDCap, a secure web-based HIPAA-compliant data collection apparatus. Data has been collected retrospectively from 2009 to 2015 and prospectively since 2015. Treatment recommendations were made based on review of all patients by a multidisciplinary SRM Conference consisting of urologists, pathologists, and radiologists. Mass size, measured by both urologists and radiologists, comorbidities, pathology results, procedure feasibility, and patient wishes were all considered in conference discussions. From this database, patients with a SK, of either congenital or acquired etiology, were included (Fig. 1).

### MWA procedure

All procedures were performed under general anesthesia. Image-guided percutaneous MWA (Neuwave™) was performed with either computed tomography (CT) guidance, ultrasound (US) guidance, or a combination, depending on tumor and patient characteristics as well as proceduralist preference. Image guidance was used for antenna placement and position confirmation. Hydrodissection was used when non-target anatomy was within the expected zone of ablation. In cases where the renal collecting system was at risk due to proximity to the antenna, the renal collecting system was cooled with continuous cold saline irrigation. After the tumor was ablated and the probes were removed, CT was performed to confirm adequate ablation. Patients were observed for 4 h after the procedure and either discharged that day or admitted for observation at the physician’s discretion.

### Data collection and statistical analysis

Patient demographics, complications, tumor diameter, and pathological results were collected. Charlson Comorbidity Index (CCI) was used to quantify comorbidities, and complications were graded using the Clavien-Dindo scale [7]. Serum creatinine was collected prior to MWA, from which estimated glomerular filtration rate (eGFR) was calculated using the 2021 CKD-EPI equation [10]. Our clinical pathway includes renal function testing and cross-sectional abdominal imaging at 6 months and annually thereafter. Contrast-enhanced MRI was preferable, and MRI-ineligible patients underwent renal mass protocol CT.

Continuous variables were summarized using medians with interquartile ranges (IQR) and categorical variables were summarized using frequency and percentage. Local recurrence free survival (LRFS) and RCC-specific metastatic recurrence free survival (MRFS) were followed from time of diagnosis to most recent abdominal imaging, last checked on August 20, 2024. Follow up imaging included abdominal CT, MRI, or renal ultrasound. Patients were censored after the most recent image or if the patient died. Overall survival (OS), RCC-specific survival (CSS), and dialysis free survival (DFS) were followed from time to diagnosis until the last chart review performed on August 20, 2024. For DFS, patients were censored if there was an additional treatment of their kidney or at death. Data was modeled with a Kaplan–Meier survival curve. Median survival time was reported for these analyses when possible, otherwise if the probability of reaching the event was less than 50% at the last time point and median could not be reported, then the mean was reported. Statistics were performed with SPSS Statistics Version 28.0.0. A Wilcoxon signed-rank test was performed (in RStudio version 2024.9.1.394) to assess the statistical difference between preoperative and postoperative eGFR values.

## Results

### Patient and tumor characteristics

A total of 26 patients with SK were included in this study, with most having acquired SK through radical nephrectomy (RN) and a smaller subset with congenital SKs (Table 1). All 26 of these patients had a form of renal cell carcinoma (RCC) on their SK, and all 26 of these patients received MWA as treatment for their RCC. The cohort had a mean age of 63.2 years (SD: 11.3) and was predominantly male (21 males, 5 females). Median CCI was 5 (IQR: 5–7). Patients were followed for a median of 28.6 months (IQR: 12.4–55.4) after treatment. Median tumor size was 2.6 cm (IQR: 2.2–3.1). Of the biopsied tumors, the predominant histology was clear cell renal cell carcinoma (ccRCC) with one case of chromophobe RCC. Of the ccRCC tumors that reported grade, 94.7% were low grade and 5.3% were high grade.

Only one patient out of 26 (3.85%) experienced a complication, which was Grade III. The patient presented two weeks following their MWA with gross hematuria and right flank pain. Imaging revealed an increase in a phlegmonous collection within Gerota’s fascia, mild hydronephrosis, and a small perinephric fluid collection. The patient was treated with ceftriaxone and underwent a right retrograde ureteropyelogram with stent placement for suspected pyelonephritis, leading to improvement in renal function prior to discharge.

**Table 1:** Patient demographics, tumor characteristics, and recurrence rates for patients with solitary kidneys treated with microwave ablation

Patient characteristics	Total (*n* = 26)
Age at Diagnosis, Mean (SD)	63.2 (11.3)
Male Sex, n (%)	21 (80.8%)
Charlson Comorbidity Index, Median (SD)	5 (2.21)
Patient Survival, n (%)	18 (69.2%)
Congenital SK, n (%)	3 (11.5%)
Tumor Grade, n (%)	
Fuhrman Grade 1	6 (23.1%)
Fuhrman Grade 2	12 (46.2%)
Fuhrman Grade 3	< 5 (< 19.2%)*
Not Reported/No Biopsy Done	7 (26.9%)
Tumor Histology, n (%)	
ccRCC	23 (88.5%)
Chromophobe RCC	< 5 (< 19.2%)*
No Biopsy Done	< 5 (< 19.2%)*
Tumor Recurrence, n (%)	
Localized	4 (15.4%)
Metastatic (from RCC)	5 (19.2%)

SK: solitary kidney, SD: standard deviation, ccRCC: clear cell renal cell carcinoma, RCC: renal cell carcinoma

*: These frequency counts smaller than 5 were redacted to protect patient privacy

### Renal function

**Fig. 2:**
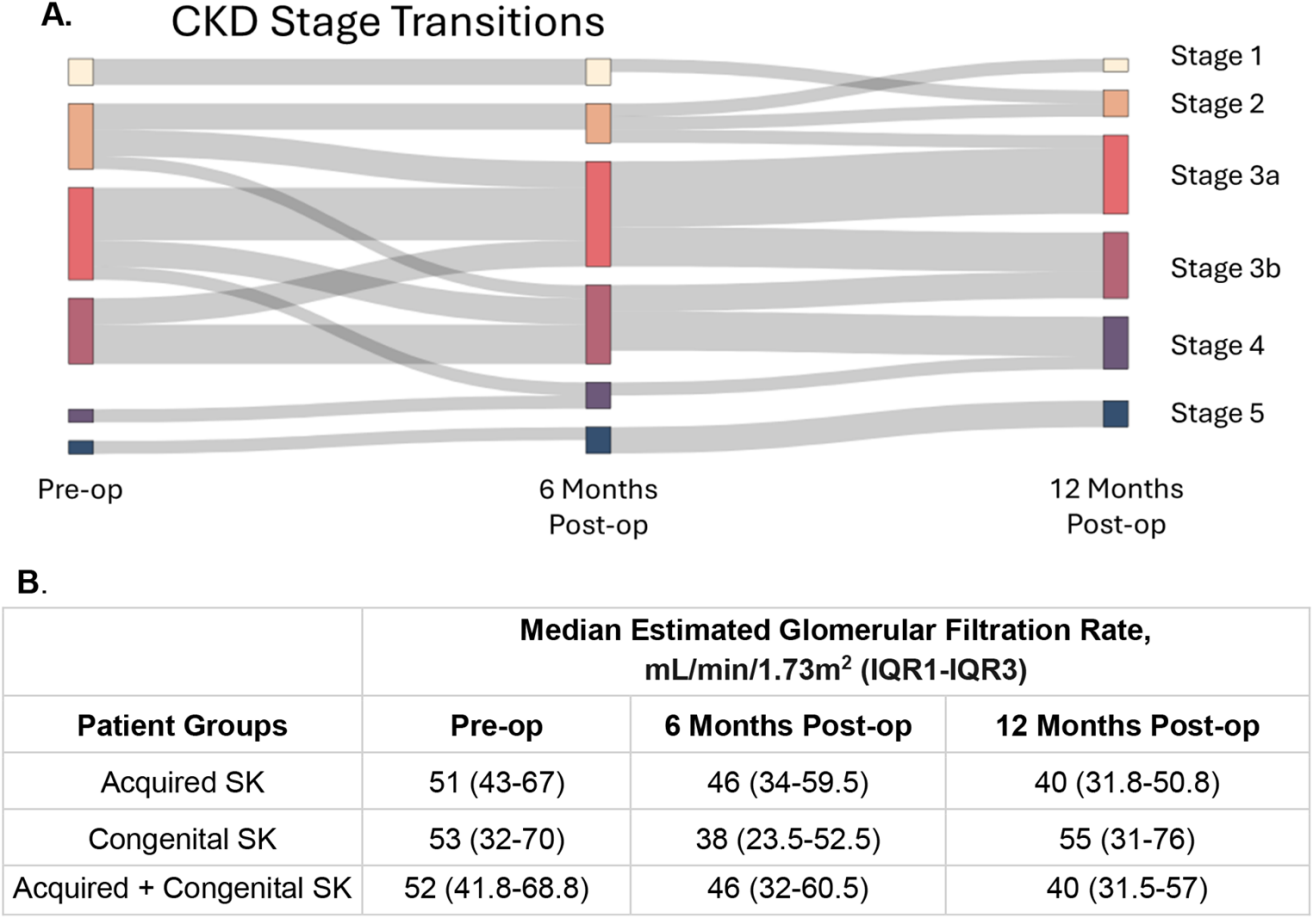
(**A**) Chronic kidney disease staging and (**B**) Median estimated glomerular filtration rate in patients prior to treatment with MWA as well as 6- and 12-months post-operatively. SK: solitary kidney, IQR: interquartile range

Chronic kidney disease (CKD) staging was evaluated preoperatively and postoperatively at 6 and 12 months (Fig. 2A). Initially, patients were mostly classified in Stage 3a (33%) followed by 2 (29%) and 3b (21%), with the fewest patients in Stages 1, 4, and 5 (8%, 4%, and 4%, respectively). Over the course of the 12-month follow-up, the proportion of Stage 3a-3b patients remained similar (33% and 24%) while there was a decrease in Stage 2 patients (10%) and an increase in stages 4–5 (19% and 10%).

Pre and post-MWA eGFR was compared in patients with acquired SK, congenital SK, and either (Fig. 2B). All patients had at least one post-op lab either at 6 or 12 months post-op. Pre-op eGFR values were acquired at a median of 12.5 days before MWA (IQR 6.5–18.8 days), and 92.3% of patients had a pre-op eGFR. Pre-op eGFR values were similar between the two individual groups at 51 (IQR 43–67) for acquired SK and 53 (IQR 32–70) for congenital SK. Overall, the pre-op eGFR was 52 (IQR 41.8–68.8) for the combined group. In each individual patient group, post-op eGFR dropped at 6 months and further decreased at 12 months. The 3 congenital SK patients had lower median eGFR at both time points compared to the acquired SK group. When analyzing both congenital and acquired SK patients together, there was a significant difference in pre-op eGFR compared to 12 months post-op eGFR (median 52 and 40, *p* = 0.0051). Of note, 84.6% and 88.5% of patients had labs at 6 months and 12 months post-op, respectively.

Four patients in the study received dialysis in the follow up period. The median time to dialysis was 98.7 months post-MWA with only one patient coming off dialysis 1.3 months after starting dialysis. Three out of the four patients on dialysis had pre-op eGFR lab values, with a mean of 70 mL/min/1.73 m [2].

### Oncological outcomes

**Fig. 3:**
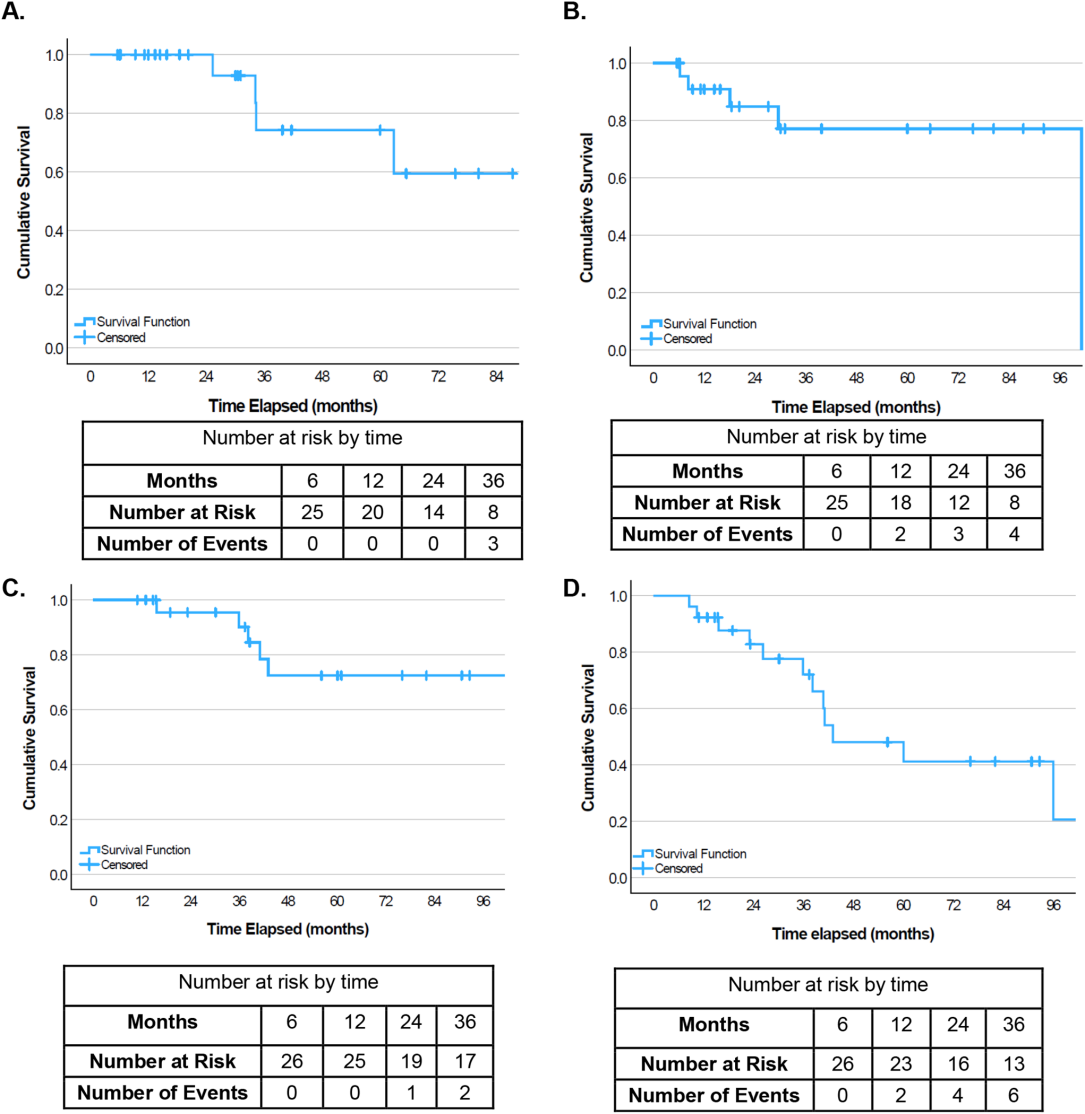
Kaplan-meier analysis of (**A**) local-recurrence free survival, (**B**) metastatic- recurrence free survival, (**C**) cancer-specific survival, (**D**) overall survival

All oncological outcomes will be described for the combined group (acquired and congenital SK). Median follow-up time was 6 months (IQR 12.4–55.4). Four patients (15.4%) experienced local recurrence. For LRFS (Fig. 3A), mean survival time was 69.4 months. There were 5 patients (19.2%) that experienced metastatic recurrence. Metastases were found in the right psoas muscle (1 patient, 20%), pancreatic tail and left psoas muscle (1 patient, 20%), small intestine (1 patient, 20%) and bone (2 patients, 40%). For MRFS (Fig. 3B), median survival time was 101.1 months. Overall, 2 patients had both local and metastatic recurrence, these were considered separately in LRFS and MRFS analyses, respectively.

There were 12 deaths overall, with all deaths in acquired SK. Of those deaths, 5 were RCC-specific, with 4 due to metastatic RCC and one patient dying of local recurrence. Confirmation of metastatic RCC in all four of these patients was done through biopsy and imaging results. For the last RCC-specific death, the patient had locally progressive RCC without evidence of widespread metastatic disease. With ECOG 3 and comorbidities including psychiatric and cardiac conditions, renal insufficiency, and protein malnutrition, medical oncology had no treatments to offer. She enrolled in hospice 6 months prior to her death. Given her last imaging showed no metastases but did show a large partially obstructing kidney mass, her death was attributed to RCC. Mean time for RCC-specific survival was 94.7 months (Fig. 3C). The remaining non-RCC related deaths were due to non small-cell lung cancer (1 patient, 8.3%), nontraumatic intracerebral hemorrhage (1 patient, 8.3%), multi-organ failure (1 patient, 8.3%), perforated colon (1 patient, 8.3%), and unknown causes (2 patients, 16.7%). Median and mean time for overall survival was 43.1 and 61.7 months, respectively (Fig. 3D). After 36 months, the OS rate decreased more rapidly compared to the CSS rate.

## Discussion

As oncological control for cT1a SRMs becomes more comparable between PN and thermal ablative measures, maintaining kidney function and preventing the morbidity of CKD becomes increasingly important. Many studies have shown that MWA can achieve excellent cancer control and is especially suited for patients who are high-risk for surgical complications [4–6]. With the low renal reserve of SK patients, avoiding renal ischemia time and achieving cancer control is the ideal balance.

We found that at 12 months post-MWA, most patients experienced a decrease in eGFR. For those with follow up at 12 months, 50% had CKD upstaging. There was a significant difference in pre-op eGFR compared to 12 months (median 52 and 40, *p* = 0.0051). Two other studies have looked at MWA in SK with cT1a SRM. Meng et al. found no significant difference in creatinine for 16 patients when comparing pre-op, 1 day post-op, and 24 months post-op [7]. Their median pre-op and 24-month creatinine was 1.06 µmol/L and 1.08 µmol/L, respectively. Lin et al. also reported that there was no significant change from pre-op (1.11 Cr) compared to most recent follow up at approximately 9.3 months [4]. Our median pre-op and 12-month creatinine was 1.3 and 1.6, which also showed no significant difference (*p* = 0.11). However, eGFR is much more sensitive to change as it takes into account patient characteristics [11]. Our patients had worse kidney function at presentation, which has been shown to be a predictor for further worsening kidney disease [12].

More broadly, it is unclear if these findings generalize to patients with two functional kidneys because few studies directly compare SK and 2-kidney patients. Our renal function findings are similar to a prior study by Qiu et al. analyzing a different cohort from the same database. Comparing eGFR at 6 months post-op vs. pre-op, our cohort had a median eGFR change of 6 (Fig. 2B) compared to an eGFR change of 5 for patients with 2 kidneys [13]. Furthermore, Ibrahim et al. found that there was no difference in renal outcomes between SK patients and age and sex-controlled patients with 2 kidneys, however, the 2 groups had different baseline comorbidities [14].

Our studies reported similar rates of CSS at 1, 2, and 3 years post-op compared to Meng et al [7]. However, their study reported no local recurrence, contrasted to our rate of 15.4% local recurrence. Furthermore, they had better OS rates at 1, 2, and 3 years post-op (100%, 93.3%, and 93.3%, respectively) compared to ours (92.3%, 84.6%, and 76.9%). The higher local and metastatic recurrence could be due to our median mass size being larger at 2.6 cm (2.2–3.1) compared to theirs at 2.1 cm (1.7–2.8), which has been shown to be correlated to recurrence in patients with nonmetastatic RCCs ≤ 4 cm [15]. Furthermore, a study by Guan et al. on patients with SK receiving MWA or PN observed similar 3-year CSS and OS rates (90.4% and 91.3%, respectively) compared to our study (92.3% and 76.9%), with our cohort again having a lower OS rate [16]. Furthermore, our 6 month, 12 month, and 36 months LRFS rates (100%, 100%, and 88.5%) were similar to a prior study on the same cohort with the majority of patients having 2 kidneys with 3–4 cm RCC (100%, 100%, and 94.5%) [13].

Our oncologic findings are comparable to studies using other ablative techniques. In a study by Xiaobing et al. comparing RFA and PN in SK with RCC, the local recurrence rate of RFA was 18.75%, similar to ours at 15.4% [17]. However, they reported a lower metastasis rate of 6.3%, compared to the 19.2% observed in our cohort. A meta-analysis conducted by Liu et al. compared the safety and efficacy of CA and PN in SK patients [18]. When looking at local and metastatic recurrence rates of CA, Liu et al. observed a local recurrence rate of 14.6% and a metastatic recurrence rate of 9.7%, compared to ours (15.4% and 19.2% respectively), with our study having a higher metastatic recurrence rate.

One explanation for our lower OS rate is that our cohort had a median CCI index of 5, which suggests a high baseline comorbidity risk. The most likely source of discrepancy in metastatic recurrence outcomes is patient selection, tumor characteristics, and duration of follow up. Our study, in conjunction with Xiaobing et al. and Liu et al.’s findings, highlights the need for further research to demonstrate these differences and broaden the potential therapeutic options for managing RCC and its associated metastatic recurrence in this high-risk patient population [17, 18].

This study is limited by the small cohort of only 26 individuals. There were three congenital SK patients included, and this may skew our results. Prior work has shown that congenital SK, but not acquired SK, can undergo compensatory hypertrophy, which could lead to differences in eGFR over time [19, 20]. Reliability of our findings is also limited by the variability in patient follow-up adherence. As such, we cannot fully assess whether MWA contributes to sustained renal function preservation or if further decline may occur beyond the 12-month period. Future research with extended follow-up durations and comparison to our gold standard of PN is necessary to demonstrate these long-term outcomes and to provide a more comprehensive understanding of how MWA affects SK patients.

## Conclusion

The purpose of this study was to evaluate the safety and efficacy of MWA for SRMs in SK patients, focusing on renal function preservation and oncological outcomes. While the overall decline in renal function post-MWA was notable, the comparable rate of local recurrence to literature values for patients with SK treated by other ablative means demonstrates that it may be a viable alternative management strategy. It may be particularly suited for high-risk SK patients with RCC where preserving kidney function is paramount.

## Data Availability

Raw data used for the analysis in this manuscript are not publicly available to preserve patients’ privacy.
